# Supplementary material for: Altered Immunomodulatory Responses in the CX3CL1/CX3CR1 Axis Mediated by hMSCs in an Early In Vitro SOD1G93A Model of ALS
Source: Biomedicines. 2022 Nov 14;10(11):2916. doi: 10.3390/biomedicines10112916 (PMC9688016; doi:10.3390/biomedicines10112916)
Supplement: Supplementary file 1 [file biomedicines-10-02916-s001.zip › biomedicines-1919764-supplementary.pdf]

Supplementary Materials

Supplementary Table S1a. Percentage of hMSCs per production process

Percentage of hMSCs per production process**Production Process**	**hMSC [%]**	**Average [%]**
V1.3	96.8	97,5
V1.4	95,5	
V1.6	97,1	
V1.7	98,3	
V1.8	99,7	

Percentage of hMSCs calculated for each production process by FACS analysis as determined by the expression of CD73, CD90 and CD105 and the absence of CD45

Supplementary Table S1b. Human specific primers for qRT-PCR analysis.

**Gene**		**Primer sequence (5´->3´) forward/reverse**	**Product length**	**cDNA concentration per reaction**	**Designed by/with**
CX3CL1	NM_002996.6	GGA TGC AGC CTC ACA GTC CTT AC	83	50 ng/well	[66]
		GGC CTC AGG GTC CAA AGA CA			
CX3CR1	NM_001337.3	TGA CTG GCA GAT CCA GAG GTT	164	50 ng/well	[67]
		GTA GAA TAT GGA CAG GAA CAC			
IL1b	NM_000576.2	AGC TCG CCA GTG AAA TGA TGG	148	5 ng/well	NCBI primer - blast
		TGG TCG GAG ATT CGT AGC TGG			
IL6	NM_000600.5	CAT CCT CGA CGG CAT CTC AG	164	5 ng/well	NCBI primer - blast
		TCA CCA GGC AAG TCT CCT CA			
IL8	NM_000584.4	TCC TGA TTT CTG CAG CTC TGT GT	104	5 ng/well	NCBI primer - blast

		TTT GGG GTG GAA AGG TTT GGA GTA			
VEGFA	NM_001025366.3 NM_001025368.3 NM_001204385.2 NM_001025370.3 NM_001171622.2 NM_003376.6 NM_001033756.3 NM_001025369.3 NM_001025367.3 NM_001317010.1	CTG CTG TCT TGG GTG CAT TG	378	5 ng/well	[68]
		TTC ACA TTT GTT GTG CTG TAG			
CNTF	NM_000614.4	TGA TTA GGC CCG CCA AAC TT	117	5 ng/well	NCBI primer - blast
		CTG GTA TAA GCC GTG CCC AA			
BDNF	NM_170733.3	AGC AGC TGC CTT GAT GGT TA	121	5 ng/well	NCBI primer - blast
		GCA GCC TTC ATG CAA CCA AA			
FGF2	NM_002006.5	GGA GAA GAG CGA CCC TCA CA	93	5 ng/well	NCBI primer - blast
		GCC AGG TAA CGG TTA GCA CAC			
GDNF	NM_000514.4	TAA TGT CCA ACC TAG GGT CTG CG	150	5 ng/well	NCBI primer - blast
		GGA GGC CTC TTA CCG GCG			
IGF1	NM_001111283.2	TGT GGA GAC AGG GGC TTT TA	245	50 ng/well	[69]
		CCT GCA CTC CCT CTA CTT GC			
IGF2	NM_000612.6	CCG CTG TTC GGT TTG CGA C	100	5 ng/well	NCBI primer - blast
		AGC ACC AGC ATC GAC TTC CC			
NGF	NM_002506.2	AGC GCA GCG AGT TTT GGC	191	5 ng/well	NCBI primer - blast
		TGC CGA TCA GAA AAG CTGT GA			

PPIA	NM_021130.5	GCCGAGGAAAACCG TGTACT	109	5/50 ng/well	NCBI primer - blast
		TGTCTGCAAACAGCT CAAAGG			
B2M	NM_004048.4	AGCGTACTCCAAAGA TTCAGGTT	306	5/50 ng/well	NCBI primer - blast
		ATGATGCTGCTTACA TGTCTCGAT			
HPRT1	NM_000194.3	CTG GCG TCG TGA TTA GTG ATG	139	5/50 ng/well	NCBI primer - blast
		TCT CGA GCA AGA CGT TCA GTC			
TUBA4A	NM_006000.3 NM_001278552.2	GGT GGG CAT CGA CTC CTA TG	71	5/50 ng/well	NCBI primer - blast
		AGT GAA TAG GCT CCA GGC AG			

Genes, human primer sequences (forward and reverse), product length, quantity (per reaction) and design of primers applied for qRT-PCR of hMSCs in hMSC-MN co-cultures.

Supplementary Table S1c. Mouse specific primers for qRT-PCR analysis.

**Gene**		**Primer sequence (5´>3´) forward/reverse**	**Product length**	**cDNA concentration per reaction**	**Designed by/with**
CX3CL1	NM_009142.3	ATT GGA AGA CCT TGC TTT GG	117	5 ng/well	[22]
		GCC TCG GAA GTT GAG AGA GA			
CX3CR1	NM_009987.4	CTG TTA TTT GGG CGA CAT TG	93	5 ng/well	[22]
		AAC AGA TTT CCC ACC AGA CC			
IL6	NM_031168.2	ACT TCC ATC CAG TTG CCT TC	80	5 ng/well	[70]
		GTC TCC TCT CCG GAC TTG TG			
IL8	NM_011339.2	TGG GTG AAG GCT ACT GTT GGC	145	5 ng/well	NCBI primer - blast
		TGT TCT CAG GTC TCC CAA ATG AAA			

VEGFA	NM_001025250.3<br>NM_001025257.3<br>NM_001287056.1<br>NM_001287057.1<br>NM_001287058.1<br>NM_009505.4	TaqMan Mm00437304_m1	77	20 ng/well	Applied Biosystems
CNTF	NM_170786.2	CTT ACG CTC CAA GTT TCT GCC T<br>CTT CTC AAA GAG GCC ACC ATC T	129	5 ng/well	[71]
BDNF	NM_007540.4	ACT GAG CAA AGC CGA ACT TCT C<br>TCT CAC CTG GTG GAA CAT TGT G	133	5 ng/well	[71]
FGF2	NM_008006.2	TaqMan Mm00433287_m1	61	20 ng/well	Applied Biosystems
GDNF	NM_010275.3	TGA CCA GTG ACT CCA ATA TGC C<br>CCG CTT GTT TAT CTG GTG ACC T	111	5 ng/well	[71]
NGF	NM_013609.3	ACA CTC TGA TCA CTG CGT<br>CCT TCT GGG ACA TTG CTA	70	5 ng/well	[72]
IGF1	NM_001314010.1<br>NM_010512.5<br>NM_001111275.2<br>NM_001111276.1<br>NM_001111274.1	TCA TGT CGT CTT CAC ACC TCT TCT<br>CCA CAC ACG AAC TGA AGA GCA T	124	5 ng/well	[73]
IGF2	NM_001315488.1<br>NM_001122736.2<br>NM_001122737.2<br>NM_010514.3	ACA ACT TCG ATT TGA ACC ACA TTC<br>GAG AGC TCA AAC CAT GCA AAC T	79	5 ng/well	[73]
GAPDH	NM_001289726.1<br>NM_008084.3	GAA CAT CAT CCC TGC ATC CA<br>CCA GTG AGC TTC CCG TTC A	78	5 ng/well	NCBI primer - blast
PPIA	NM_008907.2	TGC ACT GCC AAG ACT GAA TG<br>CCA TGG CTT CCA CAA TGT TC	85	5 ng/well	NCBI primer - blast

β-Actine	NM_007393.5	GAG CTA TGA GCT GCC TGA CG<br>AGT TTC ATG GAT GCC ACA GG	118	5 ng/well	NCBI primer - blast
HPRT1	NM_013556.2	TaqMan Mm99999915_g1	109	20 ng/well	Applied Biosystems

Genes, mouse primer sequences (forward and reverse), product length, quantity (per reaction) and design of primers applied for qRT-PCR of MNs in hMSC-MN co-cultures.

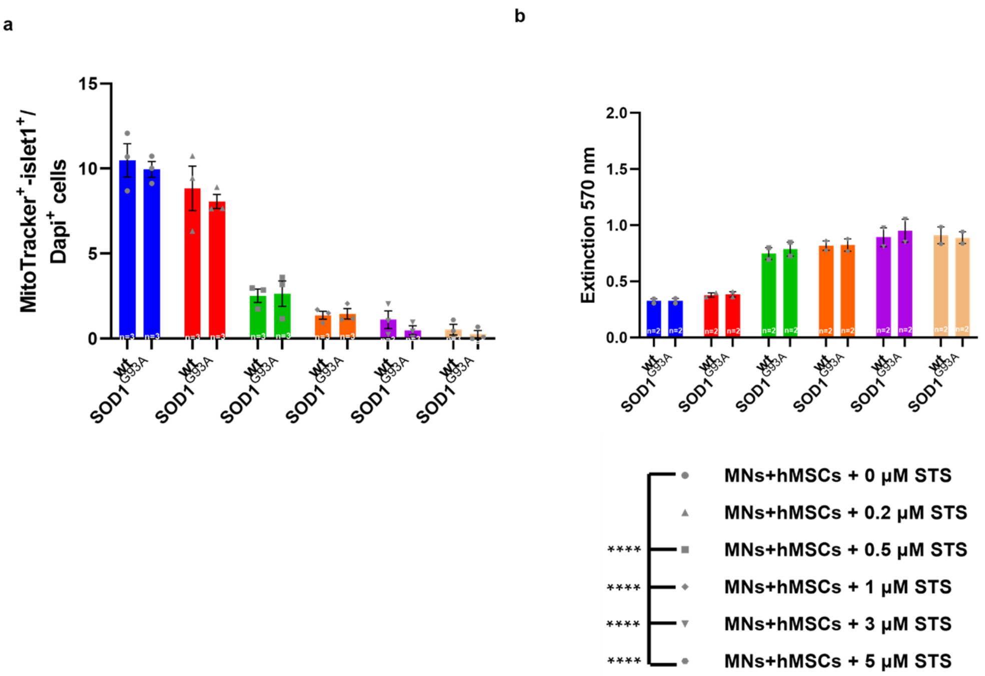

Supplementary Figure S1. Cytotoxic decrease in MN number and increase in MN-hMSC LDH levels after exposure of increasing STS concentrations. Assessment of MN number and viability in primary MN-hMSC co-cultures after STS exposure. 10,000 wildtype or $SOD1^{G93A}$ motor neurons were seeded on 10,000 hMSCs. After 5 DIV cells were exposed to increasing STS concentrations (0.2–5 µM) for 24 h. After STS exposure, on day 7, medium was removed for LDH assay and cells were stained for MitoTracker, islet1 and DAPI. **(a)** Double positive ($MitoTracker1^{+}/islet1^{+}$) cells were counted and evaluated. Significant decrease of $SOD1^{G93A}$ and wildtype MN number when co-cultured on hMSCs and exposed to a STS concentration of 0.5 µM and higher as quantified by immunocytochemistry after 7 DIV ($n = 3$). **(b)** Viability measured by LDH assay. Significant increase of lactate dehydrogenase activity in medium of $SOD1^{G93A}$ and wildtype MNs when co-cultured on hMSCs and exposed to a STS concentration of 0.5 µM and higher as quantified by LDH assay after 7 DIV ($n = 2$). All analyses were performed by repeated measurements 2-way ANOVA: $^{****}p < 0.0001$ and Tukey`s multiple comparisons post-hoc test. Single values are represented as repeated measurements together with mean ± SEM.
